# Mental health system governance in Nigeria: challenges, opportunities and strategies for improvement

**DOI:** 10.1017/gmh.2016.2

**Published:** 2016-03-16

**Authors:** J. Abdulmalik, L. Kola, O. Gureje

**Affiliations:** 1 Department of Psychiatry, College of Medicine, University of Ibadan, Nigeria; 2 Department of Psychiatry, WHO Collaborating Centre for Research and Training in Mental Health and Neuroscience, University of Ibadan, Nigeria

**Keywords:** Governance, health systems, mental health, Nigeria, policy & systems

## Abstract

**Introduction:**

A health systems approach to understanding efforts for improving health care services is gaining traction globally. A component of this approach focuses on health system governance (HSG), which can make or mar the successful implementation of health care interventions. Very few studies have explored HSG in low- and middle-income countries, including Nigeria. Studies focusing on mental health system governance, are even more of a rarity. This study evaluates the mental HSG of Nigeria with a view to understanding the challenges, opportunities and strategies for strengthening it.

**Methodology:**

This study was conducted as part of the project, Emerging Mental Health Systems in Low and Middle Income Countries (Emerald). A multi-method study design was utilized to evaluate the mental HSG status of Nigeria. A situational analysis of the health policy and legal environment in the country was performed. Subsequently, 30 key informant interviews were conducted at national, state and district levels to explore the country's mental HSG.

**Results:**

The existing policy, legislative and institutional framework for HSG in Nigeria reveals a complete exclusion of mental health in key health sector documents. The revised mental health policy is however promising. Using the Siddiqi framework categories, we identified pragmatic strategies for mental health system strengthening that include a consideration of existing challenges and opportunities within the system.

**Conclusion:**

The identified strategies provide a template for the subsequent activities of the Emerald Programme (and other interventions), towards strengthening the mental health system of Nigeria.

## Introduction

The adoption of a health systems approach to understanding and focusing intervention efforts for improving health care services is currently gaining popularity. This reflects the appreciation that interactions and synergies exist between and across the various components and sectors of the health care system (WHO, 2007; De Savigny & Adam, 2009). Thus, every intervention is likely to have system wide implications, the intended and unintended consequences of which need to be taken into consideration.

The world health organization (WHO) defines the health system as ‘all activities whose primary purpose is to promote, restore or maintain health’ (WHO, 2000). Several building blocks or components of the health system have been proposed, but common areas of agreement include leadership and governance (stewardship), resource generation, financing and service provision (WHO, 2000).

Increasing attention is now focused on the concept of health system governance (HSG), which aims at ‘ensuring that strategic policy frameworks exist and are combined with effective oversight, coalition-building, the provision of appropriate regulations and incentives, attention to system-design, and accountability’ (WHO, 2007). The goal of HSG is to encourage the establishment and strengthening of functionally strong platforms for the planning, organization and implementation of health care services in countries.

Health care governance is broadly of two types: clinical governance, which provides guidelines and principles to guide the delivery of professional services; and health system governance, which includes policy formulation, implementation and the general functioning of the health care system at national and sub-national levels. This study focuses on the latter aspects.

The dynamics of HSG is increasingly becoming an area of attention for international agencies, developmental agencies and donor bodies, especially in low- and middle-income (LAMI) countries where it can make or mar the successful implementation of health care interventions (Mikkelsen-Lopez *et al*. 2011; Bossert, 2012; van Olmen *et al.* 2012). However, despite the salience of HSG, there is a paucity of studies exploring its profile and functioning in LAMI countries, including Nigeria (Bossert, 2012; Abimbola *et al*. 2014). Studies examining mental HSG specifically in these countries are even rarer (Minas, 2012; Jenkins *et al*. 2013).

The Emerging Mental Health Systems in Low and Middle Income Countries (Emerald) is a multi-country research programme, which aims to support mental health systems strengthening in the six LAMI countries of Ethiopia, India, Nepal, Nigeria, South Africa and Uganda (Semrau *et al*. 2015). An important component of the Emerald programme is to evaluate the existing status of mental HSG in all the participating countries and to identify strategies for its improvement. The research findings here emanated from the country-specific Emerald data for Nigeria, with respect to mental health system governance.

Mental HSG considerations have become crucial in LAMI countries such as Nigeria, for a number of reasons. Firstly, the treatment gap for persons with serious mental disorders who did not receive any treatment in the preceding 12 months is estimated at 80% for Nigeria, and ranges from 76–85% for developing countries as a group (Demyttenaere *et al*. 2004). Secondly, efforts to reduce the treatment gap through the integration of mental health into primary care and the utilization of WHO initiatives such as the Mental Health Gap Action Programme – Intervention Guide (mhGAP-IG), for training non-specialists using a task sharing approach, requires an enabling policy and health system framework for it to succeed (Gureje *et al*. 2015). Thirdly, several barriers militate against the improvement of mental health systems in LAMI countries, and these are not restricted merely to the paucity of both human and material resources, but also include overarching health governance bottlenecks (Saraceno *et al*. 2007). These issues are best understood and tackled holistically using an HSG approach. Furthermore, the inter-relatedness which exists between health system performance and HSG; as well as the likelihood of improvements in either parameter resulting in a concomitant improvement in the other has been highlighted by Marais & Petersen (2015). Thus, HSG issues are crucial for any effective health system strengthening process to occur.

It is important to provide factual background context for situating health policy research, and by extension, HSG (Walt *et al*. 2008). This paper explores the mental HSG situation in Nigeria by firstly reviewing the institutional, legal and policy contexts as well as processes for the implementation of plans for service integration. Subsequently, it identifies the challenges and opportunities for strengthening the mental health system in the country.

## Methodology

**Fig. 1.**
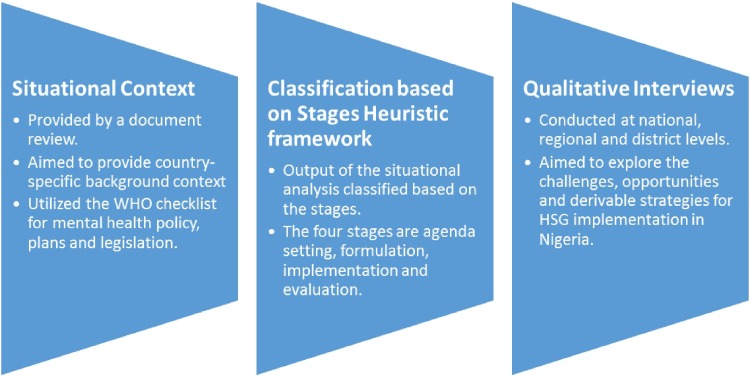
Illustration of the sequential steps of the study process.

A multi-method study design was employed to evaluate the mental HSG status of Nigeria.

(A) An initial document review was conducted as a form of situational analysis of the health policy and legal environment in the country using an adapted version of the WHO's checklist for mental health policy, plans and legislation. The documents reviewed included the National Mental Health Policy, Mental Health Plan, Mental Health Legislation as well as the Nigerian Constitution. The National Strategic Health Development Plan (2010–2015) and the broader National Health Policy were also reviewed for mental health content. Additionally, the stages heuristic framework, which breaks down the public policy process into the four stages of agenda setting, formulation, implementation and evaluation (Brewer & deLeon, 1983; Walt *et al*. 2008) were also utilized here. We recognize that the stages heuristic framework has been criticized for assuming a sequential and step-wise progression of the policy process, contrary to the often chaotic and unpredictable reality of such a process (Sabatier, 2007). Nevertheless, we believe that the framework is useful in providing a simple overview of the stages of progress in policy development and implementation. See Fig. 1.

(B) Key informant interviews (KIIs) were conducted at national, state and district (local government) levels using a questionnaire designed to explore Siddiqi's HSG framework categories (Siddiqi *et al*. 2009). The ten components of this framework are strategic vision, participation & consensus orientation, rule of law, transparency, responsiveness, equity & inclusiveness, effectiveness & efficiency, accountability, intelligence & information and ethics. Respondents were purposively identified based on their job descriptions at all three levels, indicating that they were well positioned to provide informed insight to the research questions. They were also required to complete a list of 20 capacity building needs assessment items under the following sections: mental health policy, planning and programme development; mental health systems; mental health service implementation; and mental health research. The items were rated on a five-point Likert scale ranging from a score of 1 (irrelevant to my work) to a maximum of 5 (an essential need). Additionally, probes were utilized to further explore unclear areas.

The interviews were transcribed and analysed manually using a combination of inductive and deductive coding. Predetermined codes based on the Siddiqi HSG framework were utilized. The analysis also focused on identifying the challenges and opportunities associated with each category. Emerging sub themes from the data were coded inductively, and added to the coding framework, while the original governance framework categories, were retained as parent themes. The final coded transcripts were analysed using framework analysis. The analysis was independently performed by two of the authors and subsequently harmonized by all the authors.

A compelling argument was made by Marais & Petersen (2015) about the utility of framework analysis in exploratory qualitative research, which is pertinent to this study. This form of analysis is suitable for qualitative research where categories are predetermined (with flexibility for inductive coding of emergent themes) and the major aim is to explore and describe what is occurring in a given context along the established themes (Srivastava & Thomson, 2009). Thus, it is a robust and flexible method which is also not encumbered by alignment with specific theoretical leanings (Gale *et al*. 2013).

The obvious advantage of utilizing a qualitative study approach (KIIs) across the three government levels (national, regional/state and district) is that it allowed for detailed and in-depth exploration of the various aspects of governance in a way that could provide useful insight into the perspectives of the stakeholders within the Nigerian context.

## Results

(A) *Situational analysis and document review.* The analysis of the existing policy, legislative and institutional framework for mental HSG in Nigeria revealed a complete exclusion of mental health in the key health sector documents such as the National Health Policy as well as the National Strategic Health Development Plan (2010–2015). Mental health is however mentioned in the constitution as a ground for deprivation of personal liberties via involuntary hospitalizations. There is no disability policy and no mention of mental health considerations in either the educational policy or the criminal justice policy (outside of the aforementioned involuntary hospitalizations and considerations of the Lunacy Act). Mental health is not currently captured in the routine health management information system (HMIS) of the country.

The documents which provided information about the mental health situation were the National Mental Health Policy, Legislation and Plans and the salient aspects are summarized in Table 1. The table also illustrates where Nigeria stands in regard to the stages heuristic framework.

**Table 1.** Summary content of the relevant mental health documents in Nigeria

	Theme	Baseline status	Source/evidence	Stages heuristic framework
1.1	Political support and existence of a national steering committee/task force	Yes, in existence	The National Mental Health Action Committee of the Federal Ministry of Health (FMOH)	Implementation stage
1.2	Is mental health included in the general health policy/strategic health plans?	No	National Health Policy of the FMOH and the National Strategic Health Development Plan (2010–2015)	Agenda setting stage
2	Mental health budget	No specific mental health budget, but about 3% of the health budget goes to the stand-alone neuropsychiatric facilities	WHO-AIMS Country Report (2006)	Agenda setting stage
3	Mental health policy	First passed in 1991 and revised in 2013. It recommends integration into primary care, and recognizes the need for special care for vulnerable and disadvantaged members of society. Implementation has been very poor	National Policy for Mental Health Services Delivery in Nigeria, FMOH (2013)	Implementation and evaluation stages
4	Mental health plan	First passed in 1991, and currently being revised. Poorly implemented.	The National Mental Health Programme and Action Plan for Nigeria	Formulation stage
5	Mental health legislation	A dedicated mental health legislation exists, though obsolete. Revised Mental Health Bill under consideration by the National Legislative Assembly	Lunacy laws of 1948	Formulation stage

The mental health policy of Nigeria is based on the principles of social justice and equity. Other principles include guaranteeing the rights of persons with mental, neurological and psychosocial disorders, as well as the integration of mental health care services into general health care services at all levels. It also covers mental health promotion, prevention, treatment and rehabilitation, as well as the facilitation of effective inter-sectoral collaboration (FMOH, 2013).

Health care services in Nigeria are organized into three tiers of primary, secondary and tertiary facilities. The primary health care clinics (PHCs) are manned (in decreasing order of seniority) by: registered nurses/midwives, community health officers (CHOs), community health extension workers (CHEWs) and junior community health extension workers (JCHEWs). The most senior CHO usually heads the clinic. However, when registered nurses or midwives are present, they will head such clinics. Medical doctors hardly ever feature within the primary care setting, and when they do, they serve as PHC coordinators for an entire district [Local Government Area (LGA)]. The training of the main primary care personnel in Nigeria (CHOs, CHEWs and JCHEWs) takes place in schools of health technologies where they earn a diploma in community medicine after 2 years. The PHC workers are permitted to prescribe specific and limited number of psychotropic medications (chlorpromazine, amitriptyline and phenobarbitone), but the supply of these medications are not regular.

(B) *KII results.* A total of 30 KIIs were conducted between November 2013 and April 2014, at national (*n* = 7), state (*n* = 3) and district levels (*n* = 20). Policymakers were interviewed at national and state levels (*n* = 10) while facility managers were interviewed at the district level (*n* = 20). All the policymakers approached to participate consented and were interviewed, indicating a response rate of 100%. There were 19 females (63.3%) and 11 males (36.7%). The participant profiles are presented in Table 2.

**Table 2.** KII participant profiles in Nigeria

Code	Participant	Gender	Organization
NR1	National representative	Male	Federal Ministry of Health (FMoH)
NR2	National representative	Male	Federal Ministry of Health
NR3	National representative	Male	Federal Ministry of Health
NR4	National representative	Male	Futures Group, Abuja
NR5	National representative	Male	Federal Ministry of Health
NR6	National representative	Female	NPHCDA
NR7	National representative	Female	Centre for Clinical Care & Clinical Research in Nigeria (CCCCRN)
SR1	State representative	Male	Ministry of Health, Oyo State
SR2	State representative	Male	Ministry of Health, Oyo State
SR3	State representative	Male	Ministry of Health, Osun State
DR1	District representative	Male	Department of Health
DR2	District representative	Female	Primary Health Care
DR3	District representative	Female	Primary Health Care
DR4	District representative	Female	Primary Health Care
DR5	District representative	Female	Primary Health Care
DR6	District representative	Female	Primary Health Care
DR7	District representative	Female	Primary Health Care
DR8	District representative	Female	Primary Health Care
DR9	District representative	Female	Primary Health Care
DR10	District representative	Female	Primary Health Care
DR11	District representative	Female	Primary Health Care
DR12	District representative	Female	Primary Health Care
DR13	District representative	Male	Department of Health
DR14	District representative	Male	Department of Health
DR15	District representative	Female	Primary Health Care
DR16	District representative	Female	Primary Health Care
DR17	District representative	Female	Primary Health Care
DR18	District representative	Female	Primary Health Care
DR19	District representative	Female	Department of Health
DR20	District representative	Female	Primary Health Care

The challenges and opportunities identified from the KIIs are presented below. The ten governance principles proposed by Siddiqi *et al*. (2009) were coalesced into five sections for the presentation of the results, based on the similarities and overlap of responses into: (a) strategic direction and legislation, (b) efficiency and responsiveness, (c) participation and collaboration, (d) ethics and equity and (e) intelligence and information.

### Section I: challenges of mental HSG in Nigeria

(a) *Strategic direction and legislation*. Respondents identified low government priority (90% of all respondents), poor implementation of the existing mental health policy [100% of national respondents (NR)] and an absence of desk officers for mental health at the state level [80% of NR and 100% of state respondents (SR)] as challenges to the attainment of this governance principle. The outdated mental health legislation and the slow progress with the legislative approval of the revised Mental Health Bill was another identified barrier.

The low government priority for mental health is depicted by a national representative:

*“Although mental health is important, but it is not a major killer of people. In developing countries such as Nigeria, we place more emphasis on diseases that kill people”. (NR1)*.

“*The government is more interested in communicable disease programmes such as malaria and HIV/AIDS. We don't receive any information or instructions about mental health, and it is not in our registers (data collection forms)”. (DR 5)*.

(b) *Effectiveness and responsiveness*. The identified challenges here include insufficient data to guide mental health services planning, and inadequate numbers of mental health professionals. The current figures for mental health professionals (per 100 000) in Nigeria are: 0.06 for psychiatrists; 0.19 for nurses; 0.02 for psychologists and 0.06 for other health workers (WHO, 2011). Additionally, the current training of primary health care workers does not provide them with sufficient skill to properly identify and manage mental health conditions. Others include poor funding as exemplified by the absence of a designated budget line for mental health, as well as the *ad hoc* basis of expenditure on mental health from the common purse of non-communicable diseases. Poor funding also curtails the opportunity to employ more health care personnel. The low priority for mental health and the lack of governance system considerations in financial expenditure is addressed by a facility manager:

*“Our political office holders are building physical structures rather than to equip already existing health facilities or think about issues such as (funding for) mental health.“(DR14).*”

Another district representative explains:

*“We are too few and we are overworked. Our training in primary care also has very little information about mental health so we don't feel comfortable to handle such cases”. (DR 8)*.

(c) *Participation and collaboration.* A centralized and hierarchical planning and management system is what currently operates in the Nigerian health system.

*“It is centralized and hierarchical because the chain starts from the lowest level of the head of primary health care (PHC) units, to the medical officer of health, to the state ministry of health, and finally to the federal ministry of health” (DR19)*.

The absence of formal collaborative networks and partnerships with other sectors such as education, social welfare and the justice ministries were also identified as major challenges; in addition to the limited service user and caregiver involvement in mental health.

“There are no formal collaborative partnerships with other government sectors but occasionally during health epidemics or emergencies, inter-sectoral meetings may be organized to address the issue, for example with media houses and the National Orientation Agency (NOA)”. (NR 2).

The respondents were largely (95% of all respondents) of the opinion that service users currently had limited roles in mental health care service delivery, with their role confined to participation in the clinical consultation process, rather than the planning and implementation of services. However, (65%) of the respondents were of the view that service user involvement in the planning, implementation and delivery of mental health services was feasible and would bode well for the mental health system.

*“Service users can assist in the promotion of mental health through public presentations about their personal experience with mental illness and this can help to reduce stigma.” (DR6)*.

*“They (service users) directly interact with the mental health care services system, and are therefore, in the best position to ensure that the services meet their specific needs.” (DR15)*.

(d) *Ethics and equity.* The major challenges identified here include pervasive stigma and discrimination against persons with mental illness, and by extension, the low priority accorded mental health issues by policymakers. Furthermore, while about 80% of the respondents were aware of advocacy and public health campaigns for different health programmes such as malaria and HIV, none of the respondents were aware of any advocacy or public health campaigns for mental health conditions.

*“There is little awareness about mental illness, even several health workers have poor understanding about it. Many people do not want to have anything to do with the mentally ill” (SR2)*.

*“It is those young people who are smoking cannabis in the community that are causing the increased number of mad persons. Otherwise, mental illness is not our problem in this state”. (SR3)*.

In terms of financial coverage, the National Health Insurance Scheme (NHIS) covers only about 5% of the population, and has minimal coverage of mental health conditions. Thus, the majority of health care services, including mental health, can only be accessed through out of pocket payments. Furthermore, interventions such as conditional cash transfer incentives, which exist for reproductive health programmes, are not available for mental health conditions, thus reducing financial access to care.

Ethical protection measures available for the protection of the rights of persons with mental illness include the existence of ethical review boards for research and the requirement for informed consent for participation in research by such boards.

(e) *Intelligence and information*. There is currently no routine data collection for mental health conditions in the Nigerian health care system. The absence of data for mental health conditions implies that planning decisions are taken without recourse to evidence-based considerations. Donor funded programmes for communicable diseases such as HIV/AIDS, malaria and tuberculosis appear to be the priority conditions with regular reporting systems and sometimes parallel data collection tools, in order to satisfy the requirements of the donor agencies.

There are also no routine monitoring and evaluation activities for mental health as such activities are commonly programme-driven and there are currently no mental health programmes in the public health sector.

A statement by a district representative succinctly makes this point:

*“The government is more interested in communicable disease programmes such as malaria and HIV/AIDS. We don't receive any information or instructions about mental health, and it is not in our registers (data collection forms)”. (DR 5)*.

### Section II: opportunities for strengthening mental HSG in Nigeria

(a) *Strategic direction and legislation*. Opportunities available to strengthen mental health governance include the implementation of the revised National Mental Health Policy of 2013, which has now been officially adopted by the federal government. The passage of the Mental Health Bill into law, when it occurs, will also provide another opportunity. The National Mental Health Action Committee, established by the FMOH in 2008, and which serves as a policy think tank, can provide strategic guidance and oversight to drive the mental health agenda in the country. It should also be a priority for this committee and the Mental Health Desk Officer to ensure effective integration of the mental health policy into subsequent revisions of the National Health Sector Strategic Development Plan.
(b) *Effectiveness and responsiveness*. Opportunities here include the recognition of task shifting as an effective approach for scaling up mental health care services, via the integration of mental health into primary care, as espoused in the National Mental Health Policy of the country. The adoption of the WHO's MHGAP-IG manual for training non-specialists to identify and offer basic interventions for mental health conditions in Nigeria (WHO, 2010), is the practical manifestation of this policy recommendation. The MHGAP-IG has already been contextualized and adapted for Nigeria (Abdulmalik *et al*. 2013) and the National Primary Health Care Development Agency (NPHCDA) is currently exploring its introduction into the curriculum for training primary health care workers (NR6).

Training programmes using the mhGAP-IG are currently taking place with some noticeable benefits, but facility managers would prefer a flexible training regimen and the recruitment of more staff.

*“It is very important because there are many people living with mental health problems, and we are the closest to the people at the primary health care and they will always have access to mental health care through us“(DR3)*.”

“It is very important because there are some patients who come to the hospital for malaria and what is wrong with them is not malaria but  depression, I can diagnose this correctly because of knowledge I had from Exponate” (An ongoing mental health project that involves training PHC workers to recognise and treat depression using the MHGAP-IG). (DR8)

*“There is a problem of staff shortage, such that if you take away some of them for training, it will overburden the few remaining staff. Thus flexible timing should be utilized for the trainings and more staff should be recruited. Provision of training allowances and incentives will also be helpful.” (DR14)*.

Furthermore, the strategic location of the eight neuropsychiatric hospitals across the country provides an opportunity for the effective implementation of a task-shifting approach to scaling up mental health service. These hospitals can provide training, supervision and provide specialist care at the head of the referral pathway for mental health care. Indeed, some of these hospitals already have community outreach programmes which are implemented using their own resources, both human and material.

A Mental Health Desk Officer has been appointed at the level of the Federal Ministry of Health. However, this is yet to be replicated at the regional (state) levels (NR3).

The revised Mental Health Policy recommends that a Mental Health Programme should be established and headed by an officer of at least, a Deputy Director Cadre (NR3).

(c) *Participation and collaboration*. Existing (albeit, episodic) collaborative contacts at the national level, between the FMOH and other relevant Ministries such as Information, and with professional bodies such as the Nigerian Medical Association (NMA), the Association of Psychiatrists in Nigeria (APN) as well as with Governmental Agencies such as the Primary Health Care Regulatory Agency (NPHCDA) present opportunities to be utilized.

The revised mental health policy also specifies the role of relevant stakeholders and the need for inter-sectoral collaboration. This presents an opportunity to foster greater inter-sectoral collaboration if it is implemented faithfully.

Respondents had positive views about possible gains of service user involvement in the planning, implementation and delivery of mental health services, which can be leveraged upon.

*“They can make contributions to service care development by speaking with other patients about the quality of care accessible to them.“(DR12)*.”

“Service users can assist to raise awareness about mental health through public presentations that can help to reduce stigma.“(DR6).”

*“They (service users) directly interact with the mental health care services system, and are therefore, in the best position to ensure that the services meet their specific needs.“(DR15)*.”

(d) *Ethics and equity*. Community based insurance schemes exist to reduce the burden of chronic medical conditions, including some mental health conditions, by improving financial access to care. Some states and regions also have social welfare intervention packages for vulnerable citizens such as those with mental illness.

There are pockets of advocacy and public health engagement activities in a few locations that can be strengthened and enhanced for greater visibility and impact. Lastly, ethical protection measures are available for the protection of the rights of persons with mental illness such as informed consent requirement for participation in research, and the existence of ethical review boards to review and approve research protocols.

(e) *Intelligence and information*. While there is no routine data collection for mental health conditions in the Nigerian health care system, the country has participated in large-scale epidemiological studies such as the WHO World Mental Health Surveys (Gureje *et al*. 2006), as well as the generation of WHO-AIMS country report data (WHO-AIMS, 2006), which can guide planning.

The revised mental health policy also recommends the creation of a monitoring unit to be headed by a Deputy Director that will oversee the implementation of monitoring and evaluation activities.

### Section III: capacity building priorities for mental health system strengthening in Nigeria

The mean scores of the respondents on all the items reflected their endorsement of mental health policy, planning and development; mental health systems; mental health service implementation as well as mental health research as *priority needs* (mean score of 4 out of a maximum score of 5) for which they will appreciate capacity building interventions. Only two items had a lower mean rating of 3 (*important, but not a priority need*) and these were ‘priority setting in mental health research’ and ‘human resources projection and costings’. Thus, there was a clear interest and need for capacity building interventions for mental health system strengthening activities.

## Discussion

The results presented here identify the challenges of mental HSG in Nigeria as well as the possible opportunities to address and overcome these challenges. While these are not exactly new findings, in terms of the commonly reported barriers and challenges affecting mental HSG in many other LAMI countries (Saraceno *et al*. 2007; Tumusiime *et al*. 2011; Jenkins *et al*. 2013), the unique local context issues highlighted here are useful to focus and direct intervention efforts.

Furthermore, the results provide the Emerald programme with a set of practical strategies and recommendations which will subsequently be implemented as engagement activities with relevant stakeholders with a view to promoting capacity building interventions and mental health system strengthening in Nigeria. The derived strategies and recommendations are discussed below.

## Strategic direction and legislation

*Ensuring* the effective dissemination and implementation of the revised Mental Health Policy, which has been officially adopted, such that it performs better than the previous mental health policy of 1991 should be a key priority. In this regard, the National Mental Health Action Committee (NAC) will have to fulfil its role as an expert think tank, advocacy group and implementation monitoring body to ensure that the new policy is adequately implemented.  Concerted efforts are also required to ensure that mental health is incorporated into the National Health Sector Strategic Development Plan; as well as the equivalent educational, social welfare and criminal justice system. The revised mental health policy of 2013 specifically touches on some of these aspects such as partnership with the prison services and other government agencies, but these recommendations need to be translated from policy into action.

*Strengthening* the national mental health desk officer with support and collaboration from professional bodies and organizations, as well as the encouragement of the appointment of desk officers/co-ordinators for mental health at the state levels should be pursued. The task of advocacy, engagement with stakeholders and ensuring that mental health policy receives adequate attention can be onerous for one desk officer, without the requisite support and encouragement from all stakeholders. It is a task that requires the effective mobilization of all stakeholders, especially in LAMI countries such as Nigeria (Saraceno *et al*. 2007; Walt *et al*. 2008). Furthermore, mental health policy development and implementation has now been included in the postgraduate training curriculum of psychiatric trainees in Nigeria, as well as other postgraduate courses for non-specialists. This should increase the awareness and level of requisite skills to contribute towards the process of either development or successful implementation.

*Encourage* multi-sectoral involvement in mental health policy implementation at all levels (national, regional and district) through the desk officers, and lobby for the quick passage of the revised mental health bill which is before the parliament. This is more likely to grow roots and become firmly entrenched through the establishment of structures such as inter-sectoral committees that engage and meet on a regular basis.

## Effectiveness and responsiveness

*Encourage the utilization of* the contextualized country version of the MHGAP-IG for training primary care workers, while also leveraging on the existing collaboration between the NPHCDA and the FMOH to ensure that the curriculum for training primary care workers is revised to incorporate greater mental health content. Such engagements will ensure that given the resource constraints in terms of mental health professionals in the country, the successful integration of mental health into primary care can be mainstreamed if introduced into their curriculum in the training schools for primary care workers. Emphasis should be on the acquisition of core clinical skills rather than mere theoretical knowledge, such as through supervised role – plays.

*Engage* with FMOH and State governments to ensure the national roll-out of the mhGAP-IG trainings across the country and involve mental health specialists in the provision of training and supportive supervision. This should involve not just the specialist neuropsychiatric hospitals to serve as hubs but should also include the middle-level cadre of medical officers (general medical doctors) who often serve as primary care coordinators at the district level. There is evidence for the feasibility and utility of this approach using a cascade model of delivering mhGAP-IG training in a demonstration project conducted in Nigeria (Gureje *et al*. 2015).

*Lobbying* for a budget line for mental health within the health budget at national and state levels is an important component to ensure that financial resources are made available for the implementation of mental health programmes. This should be accompanied by periodic audit of mental health expenditure, once the budget line is established.

## Information, accountability and transparency

*Develop* and integrate mental health indicators into the Integrated Support System (ISS) of the HMIS. This is an integral research objective of the larger Emerald programme, the implementation of which is on-going. This will be a crucial contribution that seeks to ensure that there is available mental health data for the country, which will facilitate evidence-based planning as well as periodic evaluation and monitoring of the mental health system performance.

Furthermore, the FMOH should produce an annual report on the mental health status of the country's population, that presents a narrative as well as illustrative figures that covers the context, inputs, processes and outcomes. This can only be feasible when there is routine data collection. Such information is particularly useful for mental health advocacy and for the monitoring of trends as well as to lobby for increased funding or services improvement.

*Ensure* compliance with the M & E directives of the MH Policy through existing civil service rules and regulations. Lastly, enhance and build upon SERVICOM's track record of monitoring quality of services and patient satisfaction.

## Participation and collaboration

*Build* on existing national collaborations with relevant ministries and other government agencies such as National Orientation Agency (NOA), NPHCDA and professional bodies; and engage with other sectors using the revised mental health policy which clearly stipulates roles and responsibilities for all stakeholders. These engagement activities should promote a broad-based coalition whose efforts should coalesce in effective mental health promotion and advocacy.

*Strengthen* existing involvement of service user groups in mental health planning and *empower* service user groups to participate in advocacy and awareness raising about mental illness through training and supportive collaborations. They should also be involved in treatment planning and decision making.

## Ethics and equity

*Encourage* the growth of community-based insurance schemes for chronic medical conditions including mental illness, to reduce the financial burden of out of pocket payments; while advocating for increased coverage of mental health conditions in the NHIS scheme.

*Engage* with the community and stakeholders to promote awareness about mental illness and reduce stigma, and support capacity building initiatives for service users and caregivers to engage with mental health services and provide feedback about quality of services.

*Enhance* public awareness campaigns with simple statements to counter widespread stigma and discrimination against persons with mental illnesses.

## Capacity building

There was a clear demand for capacity building interventions from the interviews, and this may qualify as a pre-requisite for the attainment of some of the goals such as the strategic direction and legislation; effectiveness and responsiveness; and empowerment of service users, amongst others. It is imperative to establish a clear understanding of the needs of various stakeholders; and subsequently, to plan to address such needs through targeted capacity building. This pragmatic approach for boosting health system strengthening through research, as a means of achieving universal health coverage and ensuring equitable access is now recommended (Sitthi-amorn & Somrongthong, 2000; Hanney & González-Block, 2013). It is also incontrovertible that sustainable health system strengthening cannot occur in the absence of regular capacity building initiatives to assure skills transfer (Lund *et al*. 2012; Marais & Petersen, 2015; Semrau *et al*. 2015). In line with these research evidence, Emerald activities will provide targeted interventions directed at three main categories of stakeholders: service users and caregivers; researchers and policy makers.

## Conclusion

This study contributes to the discourse of mental HSG in Nigeria by providing a comprehensive overview of the unique challenges, available opportunities and pragmatic strategies that are premised on existing realities for addressing the challenges. The mental HSG in Nigeria is weak and fraught with challenges but concerted efforts to strengthen it may start by focusing attention on some of the strategies emanating from this study. The Emerald Project will incorporate some of these strategies into its subsequent activities, in order to contribute towards strengthening the mental health systems in Nigeria. Examples of such activities include the delivery of capacity building trainings for policy makers, service users and researchers; as well as the development and pilot implementation of indicators for measuring and monitoring the performance of the mental health system in Nigeria.

A strong and functional mental HSG structure should translate into a more efficient, integrated and accessible mental health care services for the generality of Nigerians and result in a decrease in the treatment gap for mental disorders. Furthermore, we recommend similar evaluation of local HSG context, challenges and opportunities as an important first step towards developing targeted interventions for health system strengthening in a given context. While we acknowledge that such steps would require expertise that is not readily available, and will therefore not immediately translate into routine practice, nonetheless, it should be a desired goal for long-term HSG planning.
